# Factors Affecting Delivery of the HPV Vaccination: A Focus Group Study With NHS School-Aged Vaccination Teams in London

**DOI:** 10.1177/1059840518792078

**Published:** 2018-08-05

**Authors:** Lauren Rockliffe, Emily McBride, Catherine Heffernan, Alice S. Forster

**Affiliations:** 1 Research Department of Behavioural Science and Health, UCL, London, UK; 2 NHS England (London Region), London, UK

**Keywords:** human papillomavirus vaccines, immunization, delivery of health care, prevention, qualitative research, focus groups, school nursing

## Abstract

This study sought to identify barriers and facilitators to delivery of human papillomavirus (HPV) vaccination in schools. Four focus groups were conducted with 28 staff members, from four National Health Service school-aged vaccination (SAV) teams in London. Data were analyzed using thematic analysis. School engagement and support, and understanding and education about the vaccination (or conversely, a lack of) were identified as both barriers and facilitators. Limited school and team resources, fear of the vaccination, and poor consent form return were identified as barriers. Explanations for why some girls do not complete the vaccination series were provided. Individualizing approaches used to promote and encourage the vaccination was identified as a facilitating factor. Optimal delivery of the HPV vaccination program is dependent on school engagement and the allocation of time for SAV teams to promote vaccination uptake. Immunization program providers should work with schools to improve understanding and support of the HPV vaccination program.

Human papillomavirus (HPV) is a common sexually transmitted virus. Most sexually active adolescents and women contract HPV shortly after initiating sexual activity (World Health Organization, 2014). For the majority of women, the virus will spontaneously clear; however, in some cases, it can cause cell abnormalities that can develop into cervical cancer (World Health Organization, 2014). Cervical cancer is the fourth most common cancer in women worldwide (IARC, 2012), and persistent infection with HPV is known to cause almost all cases (World Health Organization, 2014).

Since 2008, a national publicly funded immunization program for HPV has been available in the United Kingdom. This program is commissioned by National Health Service (NHS) England under Public Health England (PHE) guidance and delivered by school-aged Vaccination (SAV) or school nursing teams (SAV refers to the child flu; HPV; meningitis types A, C, W, and Y; and teenage booster Section 7a immunization programs). HPV vaccination is offered to all girls aged 12–13 years (in School Year 8) and is primarily offered in secondary schools—although can also be received through community clinics or general practice—up to the age of 18 years. The vaccine is free at the point of delivery and is currently administered in a two dose series (PHE, Department of Health, NHS England, 2014). HPV immunization programs have now been implemented in 64 countries nationally, some of whom have been delivering the vaccine for over a decade, for example, United States, United Kingdom, and Australia. The vaccine is delivered through schools in 42 countries (Bruni et al., 2016). Adolescent boys are not currently included in the UK school HPV immunization program (although this is due to change in the future), but men who have sex with men up to age 45 can be vaccinated in sexual health or HIV clinics.

In the United Kingdom, uptake of the HPV vaccination is high, with 87% of 12- to 13-year-old girls receiving at least one dose in 2016/2017 and 84% of girls aged 13–14 receiving two doses (PHE, 2017a). However, there are pockets of the population who remain unvaccinated, and there is huge variability in uptake between areas; for example, uptake of the first dose in Enfield is 74%, whereas in North Yorkshire it is 98% (PHE, 2017b). More specifically, in London, uptake of the vaccination is suboptimal at 84%, placing it in the bottom 20% of areas in England (PHE, 2017b). This variation in uptake has the potential to widen inequalities in HPV-related disease.

Previous research has suggested that ethnicity may be an important factor contributing to lower uptake of the HPV vaccination. Girls from non-White British backgrounds are less likely to receive the vaccination than White British girls (Fisher, Audrey, Mytton, Hickman, & Trotter, 2014; Fisher, Trotter, Audrey, MacDonald-Wallis, & Hickman, 2013). For example, a study published in 2014 reported that 91% of White British girls had initiated the series, compared to 89% of those from Mixed backgrounds, 81% from Asian backgrounds, 79% from Chinese backgrounds, and 77% from Black backgrounds (Fisher et al., 2014). This ethnic disparity has been shown to remain even when controlling for deprivation (Fisher et al., 2014). Parents from ethnic minority backgrounds have reported concerns about the vaccine, including a lack of perceived need for it because they teach abstinence from sex before marriage to their daughters, concerns that having the vaccination may encourage promiscuity, and believing that 12–13 is too young to vaccinate their daughter (Forster, Rockliffe, et al., 2017; Marlow, Wardle, Forster, & Waller, 2009; Marlow, 2011). However, other issues affect parental decision-making, regardless of ethnicity. For example, concerns about side effects, having a lack of knowledge about the vaccination, and hearing negative stories about the vaccine from other parents have all been reported as affecting the decisions about the HPV vaccination of parents from all ethnic backgrounds (Forster, Rockliffe, et al., 2017).

While we have some understanding of the factors affecting parental decision-making, we have limited knowledge about factors that may facilitate or inhibit delivery of the HPV vaccination in the UK school context, which may subsequently affect vaccination uptake. Of the research that has been carried out in this area, interviews with nurses highlighted that commitment to the vaccination program from schools and school staff has the potential to affect uptake (Batista-Ferrer, Trotter, Hickman, & Audrey, 2016). Similarly, other studies have cited additional barriers to delivery including schools not prioritizing the vaccination and schools not following up missing consent forms or schools being unable to help organize the vaccination sessions (Batista-Ferrer et al., 2016; Brabin et al., 2011). Other reported barriers include the increasing workloads of immunization nurses and small team sizes (Brabin et al., 2011; Hilton, Hunt, Bedford, & Petticrew, 2011).

These aforementioned findings are not unique to delivery of HPV vaccination, nor to a UK setting. A recent review of studies focusing on the delivery of school based vaccination programs in high-income countries found comparable results to those previously reported (Perman et al., 2017); management and leadership of vaccination programs at a school or area level and interorganizational relationships (e.g., between education and health sectors) were found to be important factors influencing how effectively vaccination programs were delivered. More specifically, however, the review highlighted the importance of strong professional relationships and of the commitment and engagement of all school staff in influencing program effectiveness (Perman et al., 2017).

The competing demands model (Jaén, Stange, & Nutting, 1994; see Figure 1) provides a framework of interrelated factors that may create barriers to service delivery within the context of clinical preventive services. The model was designed to explain the delivery of preventive health services in primary care with a focus on physicians; however, concepts are relevant for understanding other health professionals’ ability to deliver preventive health care. According to the model, the factors that may create barriers to service delivery involve health professionals, patients, and the service environment. The model suggests that lack of time, alternative demands, and workload will affect a health professional’s ability to deliver a preventive service. Similarly, the service environment may affect delivery due to the way in which it is organized, the involvement of other (allied) health professionals, or the characteristics of the community in which the service is set. In the context of HPV vaccination, this model may help to enhance our understanding of the way in which the vaccination is delivered in schools and of the multiple competing demands placed upon immunization nurses, which may ultimately affect delivery and/or uptake of the vaccination.

**Figure 1.**
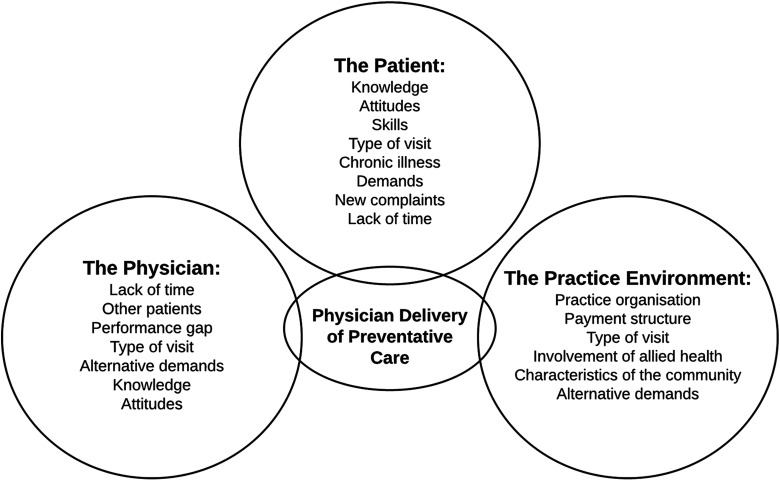
The competing demands model. *Sourc*e: Reprinted with permission from *The Journal of Family Practice*®. (Jaén et al., 1994) Feb;38(2):166–71. © 1994, Frontline Medical Communications Inc.

In the UK, there is a distinct lack of research exploring issues affecting delivery of the HPV vaccination within the school setting. Of the limited research that does exist, it has either been conducted immediately after the introduction of the vaccination, now almost 10 years ago, or not focused exclusively on the views of the immunization nurses who deliver the vaccination. Factors affecting delivery may change over time, or as a consequence of changes made to the vaccination schedule, for example, changes to the vaccine offered (from the bivalent HPV vaccine to quadrivalent vaccine) and the number of doses required (from three doses to two). Nurses’ perspectives on delivery may also shed light on ethnic and geographical disparities in uptake. It is therefore important to assess how the vaccination is being delivered at this point in time and to identify factors that may be affecting delivery of the vaccination, to better understand how uptake may be improved. The purpose of this study is to explore the barriers and facilitators to delivering the HPV vaccination within the school environment reported by immunization nurses.

## Method

This focus group study was conducted between February and September 2017 in London, UK. Ethical approval was granted by University College London (UCL) Research Ethics Committee (7427/004).

### Setting

In the UK, all routine vaccinations for school-aged children are generally delivered in school by SAV providers or school nurses. This approach has the advantage of facilitating vaccination of large groups of children at one time (with parental consent), rather than children having to individually visit a health provider, resulting in high vaccination uptake. There are 11 SAV providers in London covering 32 London boroughs. In 2016/2017, the average uptake rate for these providers for the first dose of the vaccine ranged from 79% to 89% (PHE, 2017b).

### Sample and Recruitment

Four London-based NHS SAV teams were purposively sampled. All members of the four SAV teams were eligible to participate, as we wanted participants to contribute a variety of viewpoints. The SAV teams from which participants were recruited were based in different areas of London, with varying rates of vaccination uptake, to ensure a mix of views and experiences. Recruitment was facilitated by collaborating researchers from NHS England (London) immunization commissioning team (L.R. and a public health registrar), who contacted SAV providers across London, requesting their participation in the study. Those SAV providers who agreed to participate invited their team members to take part in the focus groups on our behalf.

### Data Collection

Four focus groups were conducted with SAV team members at their place of work. We used focus groups to facilitate discussion and interaction between participants. It was not feasible to conduct more than four focus groups given the workload and time restraints imposed upon SAV teams. However, this number of focus groups was deemed sufficient, as it has been suggested that 90% of qualitative themes are likely to be discoverable within three to six focus groups (Guest, Namey, & McKenna, 2016). Each focus group comprised participants from the same SAV teams, who were therefore familiar with one another.

Focus groups were facilitated by two researchers (L.R. and a public health registrar) and took place in the participants’ workplace. All participants provided written consent, and all sessions were audio-recorded and transcribed verbatim. Focus groups lasted an average of 1 hr. Participants were also asked to complete a short questionnaire that gathered information about participants’ sex, job title, length of time in current role, and date of qualifying as a nurse/immunization nurse, if applicable.

A topic guide was used to direct the discussions and focused on the perceived barriers and facilitators to the delivery of the HPV vaccination in schools (delivery of both dose one and two). We used the competing demands model (Jaén et al., 1994) to help develop the topic guide and included prompts relating to competing factors identified in the model, where relevant. For example, prompts covered topics such as workload (related to both the health professional and service environment), and knowledge and attitudes (related to both the health professional and the patient). The researchers took detailed notes following the completion of each focus group and discussed the outcome of each session to identify ways in which the facilitation of the sessions could be improved (e.g., by improving interactions with participants).

### Analysis

Data were analyzed thematically by two researchers (L.R. and A.F.), one of whom had conducted the focus groups. Initially, these two researchers each generated codes for half of the data to develop a basic coding frame. L.R. and A.F. next discussed and refined this coding frame before using it to recode all the data using the qualitative data analysis software NVivo 11. Interpretations were made by both researchers, and any discrepancies were resolved through discussion.

The results present a summary of the themes derived from the data. Quotes are presented to illustrate the themes and are reported with focus group number and participant number. Additional participant quotes can be found in Online Supplemental Material.

## Results

### Sample Characteristics

Focus groups were conducted with a total of 28 participants and comprised between 6 and 9 participants in each group. Participants had worked in their respective roles for an average of 3 years, and most were female (*n* = 27; 96.4%). Participant job roles included nurse (17) and administrative and managerial staff, some of whom were trained as nurses; project officer (2); team assistant (2); administrator (2); clinical lead (1); operations manager (1); project manager (1); clinical director (1); and team lead (1).

### Summary of Themes

Seven main themes emerged from the data relating to issues that were perceived to either hinder or facilitate the delivery of the HPV vaccine. Lack of school engagement and support (Theme 1) was identified as a barrier to delivery, as was limited school and team resources (2), and a lack of understanding and education about the vaccination from schools, parents, and girls (3). Fear of the vaccination (4) and poor consent form return (5) were also identified as barriers. Explanations for why some girls do not complete the vaccination series (6) were provided. Conversely, facilitating factors were identified as the engagement and support of schools (1), and good understanding and education about the vaccination (3). SAV teams adopting an individualized approach (7) were also identified as a facilitating factor. Barriers and facilitators that fall under the same theme have been reported together.

### School Engagement and Support

Participants discussed the challenges of engaging schools that are unsupportive and less willing to facilitate the vaccination program, particularly larger schools, those in more deprived areas, and schools where the head teacher does not support the vaccine. Several participants found engaging certain faith schools particularly difficult; participants experienced issues such as consent forms not being handed out, alternative covering letters being attached to consent forms discouraging vaccination, and in a minority of cases, denial of entry into the school. Participants felt that in some schools, the vaccination was not prioritized due to pressures and competing demands placed upon school staff.

Participants talked about the reluctance of some schools, both faith and nondenominational, to have the SAV teams visit, and about feeling as though they were viewed as an *inconvenience*. Participants mentioned specific incidents when schools had been uncooperative by denying them the opportunity to carry out catch-up sessions, withholding parental contact information and reluctance to allow teams into the school for more time than the school deemed necessary. One participant commented that some schools merely *pay lip service* to vaccination.

What I’m finding is not all of the areas are, school-wise, supporting us. […] umm, I find that without the support of the schools, that makes it a hard job. (FG1, P4)

Conversely, the provision of support from participating schools was felt be an important facilitating factor; schools that are *on board* with the vaccination program, that are organized, and communicate well were felt to be easier to work with, and it was suggested that these factors are strongly linked to the number of returned consent forms.

At an individual-level, good working relationships between immunization nurses and key members of staff was perceived to be important, as is working with organized staff members who are persistent and proactive at “chasing up” (following up) unreturned consent forms. Furthermore, having a school nurse available (in private schools), or a coordinator, was perceived to be helpful, as was the cooperation of the school in letting immunization nurses chase up girls who have not returned their forms.

Some schools […] as our relationships gained with them, the uptake has got better and how they work in the school with getting these girls ready and getting consent forms for us has, like, increased the uptake. (FG2, P13)

### School and Team Resources

#### School resources

Allocation of school staff to assist with the vaccination was discussed by several participants who felt that this responsibility is sometimes given to staff members who are too busy to undertake such tasks such as heads of year. Furthermore, participants discussed the inconsistency of staff members with whom they liaised. Being unable to maintain a relationship with one key member of staff was perceived to be problematic; one participant explained that where good relationships did not exist, it could be difficult to encourage school staff to chase (follow up) consent forms, to organize vaccination sessions, and to release parental contact information.

There’s one school that we go into where the receptionist is the person that’s getting the children to come down for the vaccination session […] and they won’t release other members of staff to be with us during the vaccination session, so we have to allow more members of staff in that school and rely on a person who’s already very busy and stressed during that time. (FG4, P28)

#### Team resources

Participants also discussed the pressures they experienced as a team, in terms of their workload and the labor intensity of some of the tasks they have to perform. Several participants described being limited for time, which affected their ability to fully carry out certain tasks such as making phone calls to all parents who have not returned consent forms. Having few permanent staff members within the SAV teams and small team sizes were felt to compound these issues.

…doing the session is the easy bit, it’s the preparation. It’s the consent forms. It’s getting the consent forms back, and having enough time to triage, ‘cause no one can write, fill a form in properly. Y’know, they [parents] never answer questions, y’know. I spent a day last week making 70 phone calls. (FG1, P3)

### Education and Understanding

Poor education about the vaccine was cited by a number of participants as a barrier to vaccination. Firstly, it was suggested that for some parents, and schools, the importance of the vaccination was not paramount enough, which participants believed was due to a lack of understanding. Participants felt that negative parental attitudes toward the vaccination were often based on poor understanding about the nature of the vaccine. Secondly, participants gave examples of common misconceptions, which participants felt influenced parents’ decision-making. Misconceptions included parents believing that the vaccination may promote promiscuity, that girls are too young to have the vaccine, and that promoting safe sex practices or abstinence before marriage will prevent HPV infections.

Some participants felt that deprivation (a lack of material benefits considered basic necessities in society) and demographic factors affected parents’ understanding, especially in areas of London that are particularly ethnically diverse and where language barriers exist. Sources of inaccurate information (e.g., some online sources) were also highlighted by some participants as contributing toward parents’ lack of knowledge. A number of participants felt that the way in which information is delivered is also important, with accurate information sometimes hindered by poor delivery methods; for example, providing information in a lengthy format.

It’s, that’s just an education thing, isn’t it? Umm, which, I think is, is key, actually. That’s where I think there is a massive gap, not just from the parents’ point of view, but also from the schools’ point of view. I don’t think the schools really understand the importance of that vaccine as well as the parents. (FG1, P5)

Participants explained how they support parents with educational barriers to become more informed about the vaccination by providing information directly over the phone and at school open evenings, signposting parents to different sources of information and providing them with written materials such as leaflets. The importance of tailoring both the content and the delivery of information to different audiences was also discussed; the provision of culturally sensitive materials (including having materials in non-English languages) was viewed as important, as was the tailoring of delivery approaches to the demographics of different communities. Several participants discussed the benefits of phoning nonresponsive parents, as it provides an opportunity to answer questions, address misconceptions, and challenge parents’ decision-making.

And often they [parents] will say, you know, it’s good to talk rather than read the leaflet ‘cause the questions aren’t often on the leaflet that they want to discuss properly…. (FG3, P20)

Normalization and heightened awareness of the vaccination were perceived to act as facilitators to delivery of the vaccination. Knowing other girls and older siblings, who have received the vaccination, was viewed to be helpful in reducing opposition from girls. Knowing someone personally who had been affected by cervical cancer was also identified as a facilitating factor for parents, when making their decision regarding consent.

Furthermore, a number of participants discussed how good communication with girls can be used to facilitate vaccination. Participants talked about taking *any opportunity* to talk to girls about the vaccination and to educate them, as well as calming nervous girls down before the procedure, and dispelling misconceptions and fears. These approaches were discussed in addition to more formal methods of communication such as delivering talks and engaging in health promotion activities via the schools.

### Fear of Vaccination

Participants reported that some girls’ fears affected their willingness to have the vaccine. Fear was perceived to be related to the use of a needle, anticipation of pain, or in some cases the belief that the vaccine is harmful. The social impact of other girls’ negative experiences of the vaccination was perceived to heighten levels of fear; it was reported that some girls opt out after realizing other girls have done so. Some participants believed that some parents mask their own concerns about the vaccine by claiming their daughters are needle-phobic.

Some children will give us all sorts of stories that they’ve been told or they’ve heard, erm, some children will just refuse outright because they don’t want to have it done […] they’re scared…(FG2, P11)

### Poor Consent Form Return

Participants explained that parents, girls, and schools can all contribute to low rates of consent form return, which can have a direct impact on vaccination uptake. Participants felt that many parents who fail to return the forms may do so because they are too busy or time restricted rather than making an active decision to reject the vaccination. These parents may be more likely to provide consent if contacted by an immunization nurse, helping to address practical barriers. However, contacting parents can be problematic in itself, with many parents hard to get in touch with. A number of participants believed that some girls were responsible for missing forms, by not delivering them to their parents or failing to return them to school. Furthermore, disorganization within schools was felt to contribute to the problem, as was having a lack of oversight over the consent form distribution/return process within the school.

They won’t even reach home, ‘cause they [the girls] don’t wanna have it and they don’t want their parent…and if there’s no email that goes home, or anything that makes the parent aware that that’s gonna take place, then they might not even see the consent form. (FG4, P27)

### Explaining Why Some Girls Don’t Finish the Vaccination Series

A number of suggestions were made to explain why some girls receive the first dose of the vaccine but not the second. These reasons included girls being absent on the day of vaccination, having a negative reaction after the first dose (e.g., feeling unwell or developing a rash), or having a particularly negative experience (e.g., experiencing a lot of pain). Furthermore, participants cited that girls moving schools or leaving the country after receiving the first dose was a particular issue in London. Participants also felt that some parents may do more research into the vaccine after consenting to the first dose and then change their mind. Delays in delivering dose two means that on occasion delivered doses will not be included in the uptake figures, if they are done in the next school year.

I think it has to be accounted for a little bit that if the girls leave, because although we try and find out obviously where they’ve gone to, it’s sometimes out of our hands to be able to catch up with that child that’s left. (FG4, P26)

### Individualizing the Approach

Owing to differences in the ways schools work and in the varying maturity levels of girls, participants emphasized the need for individualized approaches. This was sometimes to help girls feel more comfortable with the process and included offering them biscuits or chocolate, playing music during the session, providing magazines for them to read, and allowing them to bring a friend along for moral support.

…it’s to have a nice, quiet area with, and also an area, when you’ve got the really nervous ones, where you can take them over as well, because those, y’know, don’t forget, these kids haven’t had a vaccine without their mum for years, y’know. A lot of them, y’know, they’re mature, but some of them are very immature…(FG1, P3)

Other approaches included providing incentives for the girls (e.g., bracelets, food, pens), running catch-up clinics, emphasizing the financial value of the vaccination to the girls, and feeding back to schools on their performance. Taking verbal consent was also found to be effective, and participants from one focus group felt positively about their experience of using electronic consent forms. Several participants discussed the different approaches they have used to vaccinate girls in schools, which opposed the vaccination, including gaining verbal consent from parents over the phone and taking the girls off school premises to vaccinate them.

Participants had suggestions for other approaches that they did not currently use that might improve uptake including providing schools with monetary incentives, using *name and shame* techniques with poorly performing schools and celebrity endorsement of the vaccination. Going forward, participants felt that they could have more help and support from the NHS England/PHE immunization commissioning team as well as backing from local authorities.

I personally think that if the local authorities were a little bit more driving of the programmes, I think that more of the schools would respond better because I think it’s okay it coming from us as a health issue, but it need to come from the local authority, from the education department…. (FG2, P11)

## Discussion

This study sought to understand the barriers and facilitators to delivery of the HPV vaccination in schools, using the competing demands model (Jaén et al., 1994) as a framework to guide our exploration. Our analysis of focus groups conducted with NHS SAV team members identified seven main themes relating to factors that were perceived to either hinder or facilitate the vaccination process. Engagement and support of schools was felt to affect vaccination delivery. A lack of resources within the school and within SAV teams was also perceived to act as a barrier. Education and understanding was perceived to affect parents’ attitudes and schools’ commitment to the vaccination program. Fear of the vaccination and poor consent form return were also felt to act as barriers. Participants provided explanations for why some girls do not complete the vaccination series and discussed ways in which the SAV teams individualized approaches to improve uptake.

Lack of support and engagement of schools participating in the vaccination program was perceived to be one of the main barriers to delivery of the vaccination. These findings are reflective of issues raised by school nurses in previous studies, who similarly experienced low levels of staff commitment, cooperation, and prioritization of the vaccination program (Batista-Ferrer et al., 2016; Brabin et al., 2011). Conversely, school support facilitated delivery. These findings support Perman et al. (2017) who identified that institutional relationships play an important role in the delivery of school-based vaccinations, both in the United Kingdom and abroad.

Our findings identified poor education and understanding about HPV and the vaccination as another major challenge for vaccination delivery. Previous research has shown that both girls’ and parents’ knowledge about HPV and the vaccination is associated with uptake (Bartlett & Peterson, 2011; Kessels et al., 2012), and low levels of knowledge have been found for both groups, in the United Kingdom and abroad (Allen et al., 2010; Batista-Ferrer et al., 2016; Bowyer, Marlow, Hibbitts, Pollock, & Waller, 2013; Dodd et al., 2014; Hilton & Smith, 2011; Marlow, Zimet, McCaffery, Ostini, & Waller, 2013). Our results lend support to these findings but also identify that low levels of education and understanding within the school are additional barriers to delivery of the vaccine. The lack of knowledge of school staff about the importance of the vaccination was felt to affect levels of school engagement and commitment to the vaccination program and is an issue that has not previously been identified in the literature as a barrier to delivery. Although participants believed that greater education for parents and girls would further facilitate vaccination, there is limited evidence that educational interventions for these groups are effective at improving uptake (Fu, Bonhomme, Cooper, Joseph, & Zimet, 2014). However, the use of educational interventions for school staff is a novel suggestion, as research has not previously examined the impact of education for this group on uptake of HPV vaccination. This is an area that future research could usefully explore further.

The use of individualized approaches within the school context, such as the use of electronic consent forms or providing culturally sensitive materials, may also better facilitate delivery of the vaccination. The use of incentives to encourage vaccine receipt was also an approach favored by some participants. However, there are ethical concerns associated with incentivizing vaccination (McNaughton, Adams, & Shucksmith, 2016), and therefore, incentivizing vaccination consent form return instead may be a more acceptable method. Financial incentives have shown great promise as an approach to use to encourage consent form return, as a means to increase vaccination uptake, with a recent trial suggesting that it is both practical and feasible, to offer such an incentive within the school environment (Forster, Cornelius, et al., 2017). Although individualized approaches are successfully used by some immunization teams, these are not universal approaches. Further consideration is needed about how sustainable such approaches might be and whether these can be developed into workable models of delivery.

These findings must be interpreted being mindful of the limitations of this study. The focus groups comprised team members who were familiar with one another, including senior members of staff in some cases. Although the established relationships between participants may have facilitated discussion, it is possible that some participants may have been reluctant to voice their opinions on certain topics, for fear of being judged negatively. Alternatively, some participants may have provided answers that they perceived to be socially acceptable within the group, rather than voicing their own views, and therefore creating a social desirability bias. This may also have been the case given the involvement of NHS England (London), which commissions and monitors performance of SAV services. However, focus groups were conducted by a researcher external to the NHS, and participants were reassured that their involvement in the study was confidential. There may also have been a selection bias in those who chose to participate in the study; it is possible that some of those who participated did so because they had issues or grievances that they wished to air, therefore providing a potentially skewed perspective of the issues discussed. A further consideration is that while we believe the findings of this study to be applicable and relevant to schools throughout the United Kingdom, some of the issues raised may only be of relevance to schools based in London.

### Implications for School Nurses

Our findings suggest that barriers to delivery of the vaccination program may be minimized by nurses or program coordinators attempting to educate and motivate schools regarding the importance of HPV vaccination. However, there is little evidence supporting the best approach to do this, and evaluations of approaches used by school nurses need to be documented to build an evidence base of ways to overcome barriers to delivery of the vaccination program. Efforts to improve consent form return, such as offering rewards to adolescents, may also facilitate uptake.

## Conclusions

Optimal delivery of the HPV vaccination program is dependent on school engagement and the allocation of SAV team time to promote uptake and completion of the vaccination schedule. Those providing school immunization programs should work with schools and local partners to improve understanding and support of the HPV vaccination program. Future research is warranted around the development of educational training interventions for school staff, promoting the importance of the vaccination.

## Supplemental Material

Supplementary_material - Factors Affecting Delivery of the HPV Vaccination: A Focus Group Study With NHS School-Aged Vaccination Teams in London Supplementary_material for Factors Affecting Delivery of the HPV Vaccination: A Focus Group Study With NHS School-Aged Vaccination Teams in London by Lauren Rockliffe, Emily McBride, Catherine Heffernan and Alice S. Forster in The Journal of School Nursing

## References

[bibr1-1059840518792078] Allen J. D. Othus M. K. Shelton R. C. Li Y. Norman N. Tom L. del Carmen M. G. (2010). Parental decision making about the HPV vaccine. Cancer Epidemiology, Biomarkers & Prevention, 19, 2187–2198. 10.1158/1055-9965.EPI-10-0217 20826829

[bibr2-1059840518792078] Bartlett J. A. Peterson J. A. (2011). The uptake of human papillomavirus (HPV) vaccine among adolescent females in the United States: A review of the literature. Journal of School Nursing, 27, 434–446. 10.1177/1059840511415861 21750234

[bibr3-1059840518792078] Batista-Ferrer H. Trotter C. L. Hickman M. Audrey S. (2016). Barriers and facilitators to uptake of the school-based HPV vaccination programme in an ethnically diverse group of young women. Journal of Public Health (Oxford), 38, 569–577. 10.1093/pubmed/fdv073 PMC5072158 26054910

[bibr4-1059840518792078] Bowyer H. L. Marlow L. A. V. Hibbitts S. Pollock K. G. Waller J. (2013). Knowledge and awareness of HPV and the HPV vaccine among young women in the first routinely vaccinated cohort in England. Vaccine, 31, 1051–1056. 23277094 10.1016/j.vaccine.2012.12.038

[bibr5-1059840518792078] Brabin L. Stretch R. Roberts S. A. Elton P. Baxter D. McCann R. (2011). The school nurse, the school and HPV vaccination: A qualitative study of factors affecting HPV vaccine uptake. Vaccine, 29, 3192–3196. 21354481 10.1016/j.vaccine.2011.02.038

[bibr6-1059840518792078] Bruni L. Diaz M. Barrionuevo-Rosas L. Herrero R. Bray F. Bosch F. X. de Sanjose S. Castellsagué X. (2016). Global estimates of human papillomavirus vaccination coverage by region and income level: A pooled analysis. The Lancet Global Health, 4, e453–e463. 27340003 10.1016/S2214-109X(16)30099-7

[bibr7-1059840518792078] Dodd R. H. McCaffery K. J. Marlow L. A. V. Ostini R. Zimet G. D. Waller J. (2014). Knowledge of human papillomavirus (HPV) testing in the USA, the UK and Australia: An international survey. Sexually Transmitted Infections, 90, 201–207. 24412997 10.1136/sextrans-2013-051402 PMC3995259

[bibr8-1059840518792078] Fisher H. Audrey S. Mytton J. Hickman M. Trotter C. (2014). Examining inequalities in the uptake of the school-based HPV vaccination programme in England: A retrospective cohort study. Journal of Public Health (Oxford), 36, 36–45. 10.1093/pubmed/fdt042 23620542

[bibr9-1059840518792078] Fisher H. Trotter C. Audrey S. MacDonald-Wallis K. Hickman M. (2013). Inequalities in the uptake of human papillomavirus vaccination: A systematic review and meta-analysis. International Journal of Epidemiology, 42, 896–908. 23620381 10.1093/ije/dyt049 PMC3733698

[bibr27-1059840518792078] Forster A. S. Cornelius V. Rockliffe L. Marlow L. A. V. Bedford H. Waller J. (2017). A cluster randomised feasibility study of an adolescent incentive intervention to increase uptake of HPV vaccination. British Journal of Cancer, 117, 1121–1127. 28829766 10.1038/bjc.2017.284 PMC5674104

[bibr10-1059840518792078] Forster A. S. Rockliffe L. Marlow L. A. V. Bedford H. McBride E. Waller J. (2017). Exploring human papillomavirus vaccination refusal among ethnic minorities in England: A comparative qualitative study. Psychooncology, 26, 1278–1284. 28231418 10.1002/pon.4405 PMC5599953

[bibr11-1059840518792078] Fu L.Y. Bonhomme L. A. Cooper S. C. Joseph J. G. Zimet G. D. (2014). Educational interventions to increase HPV vaccination acceptance: A systematic review. Vaccine, 32, 1901–1920. 24530401 10.1016/j.vaccine.2014.01.091 PMC4285433

[bibr12-1059840518792078] Guest G. Namey E. McKenna K. (2016). How many focus groups are enough? Building an evidence base for nonprobability sample sizes. Field Methods, 29, 3–22.

[bibr13-1059840518792078] Hilton S. Hunt K. Bedford H. Petticrew M. (2011). School nurses’ experiences of delivering the UK HPV vaccination programme in its first year. BMC Infectious Diseases, 11, 226. 21864404 10.1186/1471-2334-11-226 PMC3176210

[bibr14-1059840518792078] Hilton S. Smith E. (2011). “I thought cancer was one of those random things. I didn’t know cancer could be caught…”: Adolescent girls’ understandings and experiences of the HPV programme in the UK. Vaccine, 29, 4409–4415. 21514348 10.1016/j.vaccine.2011.03.101 PMC3176894

[bibr15-1059840518792078] International Agency for Research on Cancer (IARC). (2012). Cervical cancer: Estimated incidence, mortality and prevalence worldwide in 2012. Retrieved from http://globocan.iarc.fr/old/FactSheets/cancers/cervix-new.asp on 29/06/2018.

[bibr16-1059840518792078] Jaén C. R. Stange K. C. Nutting P. A. (1994). Competing demands of primary care: A model for the delivery of clinical preventive services. Journal of Family Practice, 38, 166–171. 8308509

[bibr17-1059840518792078] Kessels S. J. Marshall H. S. Watson M. Braunack-Mayer A. J. Reuzel R. Tooher R. L. (2012). Factors associated with HPV vaccine uptake in teenage girls: A systematic review. Vaccine, 30, 3546–3556. 22480928 10.1016/j.vaccine.2012.03.063

[bibr18-1059840518792078] Marlow L. A. V. (2011). HPV vaccination among ethnic minorities in the UK: Knowledge, acceptability and attitudes. British Journal of Cancer, 105, 486–492. 21829204 10.1038/bjc.2011.272 PMC3170970

[bibr19-1059840518792078] Marlow L. A. V. Wardle J. Forster A. S. Waller J. (2009). Ethnic differences in human papillomavirus awareness and vaccine acceptability. Journal of Epidemiology and Community Health, 63, 1010–1015. 19762455 10.1136/jech.2008.085886 PMC3960938

[bibr20-1059840518792078] Marlow L. A. V. Zimet G. D. McCaffery K. J. Ostini R. Waller J. (2013). Knowledge of human papillomavirus (HPV) and HPV vaccination: An international comparison. Vaccine, 31, 763–769. 23246310 10.1016/j.vaccine.2012.11.083

[bibr21-1059840518792078] McNaughton R. J. Adams J. Shucksmith J. (2016). Acceptability of financial incentives or quasi-mandatory schemes to increase uptake of immunisations in preschool children in the United Kingdom: Qualitative study with parents and service delivery staff. Vaccine, 34, 2259–2266. 26979137 10.1016/j.vaccine.2016.03.009

[bibr22-1059840518792078] Perman S. Turner S. Ramsay A. I. Baim-Lance A. Utley M. Fulop N. J. (2017). School-based vaccination programmes: A systematic review of the evidence on organisation and delivery in high income countries. BMC Public Health, 17, 252. 28288597 10.1186/s12889-017-4168-0 PMC5348876

[bibr23-1059840518792078] Public Health England. (2017a). Human Papillomavirus (HPV) vaccination coverage in adolescent females in England: 2016/17. Retrieved January 2018 from https://www.gov.uk/government/uploads/system/uploads/attachment_data/file/666087/HPV_vaccination_coverage_in_adolescent_females_in_England_2016_to_2017.pdf

[bibr24-1059840518792078] Public Health England. (2017b). Annual HPV vaccine coverage in England 2016 to 2017: By local authority, local team and area team. Retrieved January 2018 from https://www.gov.uk/government/uploads/system/uploads/attachment_data/file/666083/HPV_16_17_webtables.xls

[bibr25-1059840518792078] Public Health England, Department of Health, NHS England. (2014, May 14). Joint letter on HPV vaccine schedule change. Retrieved October 2017 from https://www.gov.uk/government/uploads/system/uploads/attachment_data/file/310958/HPV_Joint_Letter_14_May.pdf

[bibr26-1059840518792078] World Health Organization. (2014). Comprehensive cervical cancer control. Retrieved from http://apps.who.int/iris/bitstream/handle/10665/144785/9789241548953_eng.pdf;jsessionid=4999BBD0FC89387DB3462F9353CD3C34?sequence=1 on 29/06/2018 25642554

