# Supplementary material for: Factors Affecting Delivery of the HPV Vaccination: A Focus Group Study With NHS School-Aged Vaccination Teams in London
Source: J Sch Nurs. 2018 Aug 5;36(2):135–43. doi: 10.1177/1059840518792078 (PMC7323732; doi:10.1177/1059840518792078)
Supplement: Supplementary_material - Factors Affecting Delivery of the HPV Vaccination: A Focus Group Study With NHS School-Aged Vaccination Teams in London [file Supplementary_material.pdf]

School engagement and support	
Barriers	*"It was very like unfriendly, not very supportive and helpful, and then for the Year 9 and Year 11s, yeah, they were nice, helpful. So, you knew they didn't want us in there for the Year 8."* (FG3, P21)<br><br>*"I think some of the faith schools, erm, are quite reluctant to have us there in the first place, and the... the uptake is fairly low in those schools."* (FG4, P28)<br><br>*"…for instance, this one school that we asked, umm, "Can we do the catch-up?" We wanted to talk to the girls, why they didn't bring their consent form in, do they want to have it? And they said "No. You came in once, they had the forms, they had the opportunity. No." So, they stopped us dead."* (FG1, P4)
Facilitators	*"I think if the welfare person is repeatedly reminding young people and sending reminders out via email to parents. That seems to have an effect […] So it is possible if you've got a proactive person within the school chasing it, but it does depend on that."* (FG4, P28)<br><br>*"I think communication, just constant communication with the schools because if they know what's going on then, and you've got them on board, then it, it does make things a lot easier."* (FG3, P20)<br><br>*"I think yeah, the schools that are like organised and on board with us, you definitely, that had a direct effect on how many forms we got back."* (FG3, P21)
**School and team resources**	
Barriers	*"…in the beginning we used to go to a school, we would give a talk to the children, we'll give a talk for the teachers and then it's a different teacher who was actually arranging the session, so, you know, the importance of what we're trying to achieve is lost on some teachers*." (FG2, P11)<br><br>*"I personally find when it's passed on to a head of year, that's when, when the issue lies. Because the head of year hasn't got the time to change the consent forms, erm, like, erm, an administrator or actual school nurse would have to do."* (FG2, P3)<br><br>*"I mean, we have not been calling each one of them who hasn't returned the form. […] and that's, I guess it's, it could, it could be put down to time. If we, if we had more time, we probably would."* (FG3, P18)<br><br>*"…all of us made the packs. We had to deliver the packs [consent forms and information], so that's two hours to for us to get to [borough] to deliver the packs, then spend time trying to park to deliver the packs, so, you're out a whole day, just delivering packs. So, coming back and then we've gotta sort out another time to collect the packs. It's little things like that, really, that sort of, y'know, put a lot of pressure on the admin and all the staff, y'know, before you even get into the school."* (FG1, P3)
**Education and understanding**	
Barriers	*"Some parents don't really know the importance of their children having HPV. So, they don't return their form, and at times, when they will return the form, they don't fill it correctly. So, err, there's, a bit of, err, problem with parents' understanding of what HPV's about…"* (FG1, P7)<br><br>*"But I do think it's to do with education because a lot of these teachers and people that organise the sessions now in the schools are teachers because of the cuts that they've had in the schools, and I don't think they understand the importance of the vaccinations, and why their children are having them, because no one's there to actually explain to them what it is."* (FG2, P13)<br><br>*"…it's better to educate people, umm, and I think that that's where we're missing a bit of a trick, because we're not doing it in the right way. It's all too lengthy, and too much information out there, which causes a bit of panic, and a bit of hysteria."* (FG1, P5)
Facilitators	*"…so we always try and support the parents to try and get as much information as they can."* (FG3, P22)<br><br>*"Obviously, if parents telephone us, then we can give information over the phone and we have our contact details in the letter and the consent form that goes out through schools to parents. So they're always welcome to... To call us and quite a few parents do."* (FG4, P28)

**Education and understanding (continued...)**	
	"Yeah, they do. And it's a good thing, because once they start doing it then the next year they're more likely to do it, 'cause they all talk to each other in that community." (FG2, P11)<br><br>*"Yeah, again, you know, trying to, any, any opportunity that you, when you talk to these young people in schools it can also be just in the school and they come and ask 'cause they're curious…"* (FG3, P18)<br><br>*"So we just sort of educate them, and there has been... [name] did go in last year, didn't you, and delivered talks and stuff, which was helpful 'cause you found a lot of schools who had the talks were more understanding…"* (FG4, P26)
**Fear of vaccination**	
Barriers	*"They're scared, but in all the time I've only really heard one child say to me that she didn't want to do it because it's gonna do something bad to her."* (FG2, P11)<br><br>*"It's more about the needle phobia as well, isn't it? I think they're sort of scared of the needle rather than the vaccine itself."* (FG4, P23)<br><br>*"…another one that comes out all the time, it's 'Oh, my child's needlephobic, they won't have it done at school,' when actually, I think a lot of that comes down to the parents […] It's the parents' anxiety about their child having something at school, and, y'know, having a funny turn, or whatever."* (FG1, P8)
**Poor consent form return**	
Barriers	*"There are loads of reasons yeah, 'I haven't had the form', 'I know I should've filled it in but I didn't get time', erm, 'oh I'm so busy I just can't fill in these things', 'I couldn't get it back on time', er, yeah, there's lots of different reasons…"* (FG2, P11)<br><br>*"'Cause, the nurses can phone and phone, and phone and phone; parents don't call back […] Y'know, so that's one, that's one of the issues that I hear from, y'know, the various teams across London, umm, that is parental contact is actually very difficult."* (FG1, P5)<br><br>*"Yeah, I think it's down to the organisation of the school concerned and how they deal with the forms when they come back […] We have had on occasions situations whereby the forms that were brought in by the young people from the parents who have signed it has gone to the form tutor and somewhere along the line it doesn't get given to the person who's responsible for those forms in the office situation."* (FG3, P16)
**Explaining why some girls don't finish the vaccination series**	
Barriers	*"And also you'll get girls that just, they've had one, and then they come for that second one and they're so nervous 'cause they remembered what the first one was like, they'll refuse point-blank for you to give it to them."* (FG3, P20)<br><br>*"…when they feel unwell after the vaccine, or they'll... Like the girls talk... You know, talk afterwards and then the parents are looking at Google and all the negative stuff that's said about the vaccines."* (FG4, P24)<br><br>*"I think sometimes you don't always capture the figures. We sometimes do dose one late so we can't get dose two captured in the figures."* (FG2, P11)
**Individualising the approach**	
Facilitators	*"…what I do, sometimes when I try and talk them into it, I tell them how expensive it is 'cause it makes people feel valued that they're being given such an expensive vaccine."* (FG3, P18)<br><br>*"So, the first class that brings 100% back of their forms gets Millie's cookies […] So, in some areas it's really worked quite well."* (FG1, P4)

Individualising the approach (continued…)	
	"Y'know, we've tried everything, we've had music in the background, put a bit of music on, we've had magazines for them to read ten minutes after, so they're all chilling, and you can't get rid of them…" (FG1, P3)<br><br>*"Could they get an email with an electronic attachment or something as a consent form rather than being something that can get lost or just doesn't reach home?"* (FG4, P27)<br><br>*"They need to get, sort of, er, pop stars […] yeah, to sort of promote it. You know, have a more positive light that the young girls can identify with…"* (FG4, P23)<br><br>*"It would be quite good if we could have the literature in other languages, and specific dialects within the language."* (FG2, P11)
